# Portable Drowsiness Detection through Use of a Prefrontal Single-Channel Electroencephalogram

**DOI:** 10.3390/s18124477

**Published:** 2018-12-18

**Authors:** Mikito Ogino, Yasue Mitsukura

**Affiliations:** 1 Dentsu ScienceJam Inc., Akasaka, Tokyo 107-0052, Japan; 2 School of Integrated Design Engineering, Keio University, Yokohama, Kanagawa 223-8522, Japan; mitsukura@keio.jp

**Keywords:** electroencephalogram, single-channel, drowsiness, Karolinska sleepiness scale, portable system, support vector machine, power spectral density

## Abstract

Drowsiness detection has been studied in the context of evaluating products, assessing driver alertness, and managing office environments. Drowsiness level can be readily detected through measurement of human brain activity. The electroencephalogram (EEG), a device whose application relies on adhering electrodes to the scalp, is the primary method used to monitor brain activity. The many electrodes and wires required to perform an EEG place considerable constraints on the movement of users, and the cost of the device limits its availability. For these reasons, conventional EEG devices are not used in practical studies and businesses. Many potential practical applications could benefit from the development of a wire-free, low-priced device; however, it remains to be elucidated whether portable EEG devices can be used to estimate human drowsiness levels and applied within practical research settings and businesses. In this study, we outline the development of a drowsiness detection system that makes use of a low-priced, prefrontal single-channel EEG device and evaluate its performance in an offline analysis and a practical experiment. Firstly, for the development of the system, we compared three feature extraction methods: power spectral density (PSD), autoregressive (AR) modeling, and multiscale entropy (MSE) for detecting characteristics of an EEG. In order to efficiently select a meaningful PSD, we utilized step-wise linear discriminant analysis (SWLDA). Time-averaging and robust-scaling were used to fit the data for pattern recognition. Pattern recognition was performed by a support vector machine (SVM) with a radial basis function (RBF) kernel. The optimal hyperparameters for the SVM were selected by the grind search method so as to increase drowsiness detection accuracy. To evaluate the performance of the detections, we calculated classification accuracy using the SVM through 10-fold cross-validation. Our model achieved a classification accuracy of 72.7% using the PSD with SWLDA and the SVM. Secondly, we conducted a practical study using the system and evaluated its performance in a practical situation. There was a significant difference (* *p* < 0.05) between the drowsiness-evoked task and concentration-needed task. Our results demonstrate the efficacy of our low-priced portable drowsiness detection system in quantifying drowsy states. We anticipate that our system will be useful to practical studies with aims as diverse as measurement of classroom mental engagement, evaluation of movies, and office environment evaluation.

## 1. Introduction

Recently, researchers have tried to identify human mental states, such as drowsiness, stress, and concentration, by electroencephalogram (EEG) [1,2]. EEG is a method of measuring the electrical activity of the brain by adhering electrodes to the scalp. For drowsiness, the EEG alert/sleep detection system has been studied in areas as diverse as driver safety, classroom rating in schools, and evaluation of workplace environments [3,4].

Previous studies have demonstrated that EEG can detect drowsiness with over 80% accuracy [5,6,7]. Machine learning and pattern recognition approaches make it possible to detect human drowsiness in real time [5,8]. By using multichannel EEG devices, information-rich high-quality data can be obtained and a high accuracy of drowsiness detection can be attained. However, such devices are difficult to attach to the scalp of users, and application of the gel required for usage of the electrodes is time-consuming. Moreover, the cost of conventional devices is high. Therefore, for use in practical situations, such as neuro-marketing and educational fields, a low-priced and portable device is needed for efficient and cost-effective data collection. In this study, we aim to develop a drowsiness detection system that can be used with a low-priced and portable device. We used Mindwave Mobile, which makes use of two dry electrodes and Bluetooth to release subjects from the constraints of conductor cables. The cost is much cheaper than conventional EEG devices because the number of electrodes and their required locations are limited (Fp1–A1). Moreover, the EEG signal detected from the limited locations can be further used for the detection of prefrontal brain activity related to human experiences such as emotion [9,10,11]. Despite these advantages, the restrictions imposed by our method make drowsiness detection more difficult in comparison with other studies that use single-channel EEG [12,13,14]. Over the course of this study, we demonstrate that human drowsiness can be accurately detected using this constrained but useful device. Although conventional multichannel EEG devices are more accurate in the case of drowsiness detection, usage of the low-cost device investigated in this paper may be more realistic in particular situations.

Most recent studies on drowsiness detection have focused on human states while driving [7,11]. Detecting driver vigilance has direct repercussions for safety-related businesses. However, an approach to recording human drowsiness levels using EEG outside of driving situations is necessary for application of the system to practical situations, such as classroom or workplace environments. The Karolinska Sleepiness Scale (KSS) is a well-established measure for assessing daytime drowsiness using subjective scales [15,16,17]. Similar to visual analogue scales and Likert scales [18], in the KSS, subjects select a score from 1 to 9 that best reflects their level of sleepiness: (1 = very alert, 9 = very sleepy). Kaida et al. collected EEG data and KSS scores while subjects either stared at a small postcard on the wall with open eyes or sat in the same position with closed eyes. They found that the KSS is correlated with alpha and theta EEG activity when the eyes are open [19]. Other studies have reported similar results using the KSS and EEG [20,21,22]. The KSS has also been used for scoring work and study performance in the fields of clinical and research psychology [23,24]. Hence, we used the KSS for scoring the human drowsiness state. Combining EEG and KSS to develop a drowsiness detection system, Muhammad et al. demonstrated that the states of alertness and drowsiness in driving can be discriminated using EEG and electrooculogram (EOG) [25]. These states can be related to KSS scores and classified by a support vector machine (SVM) with 76.36% accuracy using only EEG. Zhang et al. also made use of an SVM for classifying two vigilance states, alertness and drowsiness, in drivers. In this experiment, they used an SVM with power spectral density (PSD) and obtained an accuracy of over 90% [11]. According to this research, the support vector-related methods are, even in recent years, still proving to be powerful tools. Therefore, we used the SVM as the classification method in this study.

For PSD, alpha and theta bands are known to correlate with drowsiness [1,26,27]. However, we presume that other frequency ranges have the possibility of contributing to the accuracy of drowsiness detection. Therefore, we divided and regrouped frequencies more finely in this study. As the number of frequency features increases, so does the difficulty in selecting meaningful features from that data. In order to select relevant features, either the round-robin method or an optimization method, like the genetic algorithm (GA), can be applied. However, these methods require evaluation functions related to the accuracy of the classification methods, such as SVM. To compensate for the high calculation cost of these methods, we used step-wise linear discriminant analysis (SWLDA), which selects features by using feature distributions rather than classifiers. The method has been widely used for brain–computer interfaces (BCI) [28,29]. Using SWLDA greatly reduces calculation cost while still allowing for feature selection prior to the outcome of the SVM. This allowed us to allocate more time to hyperparameter selection, of which the SVM requires two: *C* and γ. We used the SVM at the level of drowsiness classification and selected the appropriate hyperparameters using the grind search [30,31].

Although PSD is a well-known feature used for drowsiness detection, the autoregressive (AR) model and entropy-based methods have also been used for fatigue detection or sleep stage classification in recent studies [32,33,34]. Therefore, we compared these features with PSD to determine the best features for usage in the development of a single-channel drowsiness detection system. Each of the aforementioned features extracts characteristics from time-series data differently. The AR model has the advantage of extracting better spectral resolution for short data segments compared with PSD. Entropy is a nonlinear parametric method which quantifies the complexity of a time-series signal. Entropy can evaluate nonlinear, unstable, and dynamic EEG signals [32]. Single-channel EEG yields less information than multichannel EEG, hence, we computed multiscale entropy (MSE) as a feature in this study.

To assess whether the drowsiness detection system can be applied within practical studies and businesses, a method of evaluation needs to be established through determination of standard criteria. There are two criteria appropriate for our evaluation: classification accuracy in the case of offline analysis and significance of the difference between conditions in practical experiments. Classification accuracy has previously been used for assessing the performance of pattern recognition classifiers, such as the SVM. In our study, we made use of 10-fold cross-validation to calculate classification accuracy, with consideration given to the overfitting characteristic of pattern recognition methods. A classification accuracy of 70% is the cutoff often explicitly or implicitly chosen as the threshold value for binary classification problems in BCI [35,36]. Using these criteria, we divided our EEG data set into two classes (alert/drowsy) and calculated classification accuracy. Although there is an established criterion for classification accuracy, it is unclear whether a system attaining this goal can be used practically in business and marketing. Therefore, we additionally calculated the significance of the difference between our conditions, a method that has been widely applied in the evaluation of practical experiments, such as sleep studies and neuro-marketing [19,37,38]. By considering the significance of the difference between conditions in a practical experiment, we ensured that our model maintains a suitable level of classification accuracy outside of model development conditions.

This paper is organized as follows. Section 2 describes the model used to estimate the drowsiness level from EEG data. This section also includes a comparison of PSD, AR modeling, and MSE, along with the classification performance results. The section goes on to demonstrate that our chosen method used for our drowsiness detection system has the best performance of the methods compared. Section 3 presents an application of the developed model in the form of a practical experiment. The results of the experiment, in which participants took part in either a drowsiness-evoking task or a concentration-needed task, are presented. The significant difference in the drowsiness estimates of our model between the two conditions serves to demonstrate that our model can correctly classify level of drowsiness. Section 4 discusses the results of these evaluations and Section 5 presents our conclusions.

## 2. Model Development

### 2.1. Materials and Methods

#### 2.1.1. EEG Recordings

**Figure 1:**
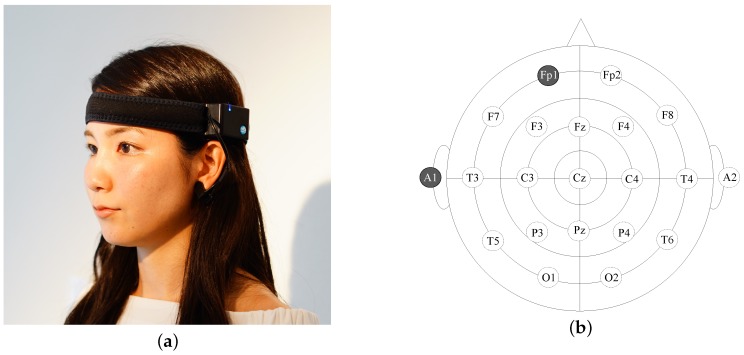
(**a**) The portable electroencephalogram (EEG) device makes use of two electrodes, one of which is positioned on the left prefrontal region (Fp1) and the other of which is positioned on the left earlobe (A1). EEG data are transferred to a smartphone via Bluetooth. The portable device uses a lithium-ion rechargeable battery; (**b**) the international 10–20 system and the measurement points used by our device (Fp1–A1).

We used a single-channel EEG device (MindWave mobile BMD version, Neurosky Inc., San Jose, CA, USA) shown in Figure 1a. The device transfers the EEG measurements to a smart device via Bluetooth. We used an iPad Air2 (iOS 11.4.1, CPU:Apple A8X, RAM:2GB) as a recording device. The sample rate of the EEG device used was 512 Hz. The data were saved by an iOS application we developed. A 50 Hz notch filter was applied by the EEG device. The dry electrodes of the device were placed on the fixed locations Fp1 and A1 in accordance with the international 10–20 system depicted in Figure 1b [39]. The active electrodes used were composed of sintered Ag/Ag-Cl material. We selected our EEG device on the basis of its price, which is around 100 USD, making it cheaper than other devices on the market. Although Fp1 is known as a suboptimal location for detecting human drowsiness, EEG measurements taken at Fp1 have been demonstrated to be correlated with drowsiness [40]. Furthermore, it is well-established that frontal area activity correlates with human emotion and stress [41,42]. When drowsiness can be estimated using the measurements from Fp1, the EEG can be used to infer other mental states of the user. Finally, the signal quality from the frontal area is better than that of other locations because the electrodes can be attached to the scalp directly without hair disturbance.

#### 2.1.2. Experimental Procedure

The experiment was approved by the Ethical Review Committee of Dentsu ScienceJam Inc. (approval number 005). Twenty-nine healthy subjects took part in our experiment (13 females and 16 males, mean age = 30.9 years, SD = 11.6). Subjects did not have any mental diseases or psychological disorders. All subjects were non-smokers and were asked to abstain entirely from intake of caffeine, nicotine, and alcohol for the duration of the experiment. We asked them to provide recordings from the EEG three times a day for 7 days. On the first day, we instructed them on how to use the EEG device and the iPad application. We explained the details of the experiment so that the subjects had a full understanding of the procedure. Before recording commenced, subjects completed an informed consent form. We made it clear that they had the right to withdraw from the experiment at any time if it caused them any discomfort.

**Figure 2:**
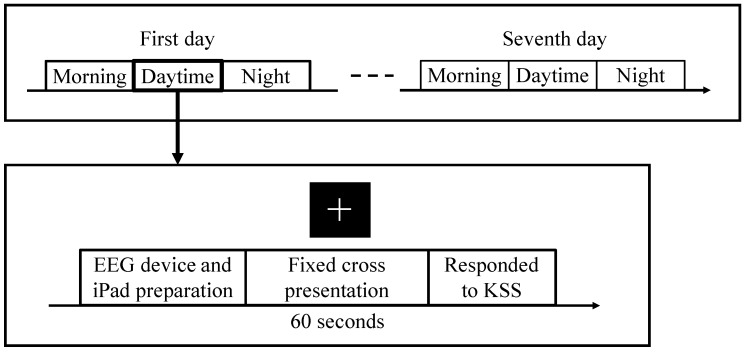
The experimental procedure, which participants repeated for 7 days. EEG recordings were obtained three times a day. A fixed cross was presented during measurement.

The EEG recordings were conducted following the procedure outlined in Figure 2. We requested that participants obtain EEG recordings three times a day: once in the morning (when they first wake up), once during the daytime (12:00–14:00), and once at night (before going to sleep). This allowed us to obtain data from both drowsy and wakeful states. Specifically, we instructed our participants to take an EEG recording within 5 min of waking up in the morning and 5 min before closing their eyes at night. At the beginning of the measurement, they attached the EEG device and prepared the iPad application. In order to equalize the effect of posture, they were instructed to sit on a chair. After preparation was complete, the application would automatically present a fixed cross image. Participants had been instructed to gaze at it during the recording while having their eyes open. EEG recording continued for 60 s. To aid in tagging the EEG data with labels, we prepared a Japanese version of the KSS, which comprised a 9-point Likert scale asking participants to rank their drowsiness [15,17]. Participants responded to the KSS after each EEG recording. In order to obtain a data set representing a range of drowsiness levels, we requested that participants conduct measurements at least once while feeling drowsy during the day.

#### 2.1.3. Denoising and Artifact Removal

The EEG signal can be corrupted by noise in the range of 50 Hz or 60 Hz. Eye blinking and muscular artifacts can also disrupt the interpretation of the EEG signal. Cutting out the signal with these noise ranges is not difficult, but it results in the removal of necessary information. To retain characteristics of the EEG signal while decreasing noise and artifacts that may complicate interpretation, in recent years, wavelet-based denoising and removal methods have been used [43,44]. In our study, we decreased the noise from the 50–60 Hz range through wavelet-based denoising with soft thresholding [45]. A wavelet transformation was applied to produce wavelet coefficients and the noise was reduced following application of the soft threshold algorithm. After the denoising step, muscular and eye blink artifacts were eliminated. These artifacts are present on the frequencies of 8 Hz and 40 Hz [44]. After applying wavelet transformation to the EEG signal, a threshold-based method was used to eliminate the frames of the second and fourth coefficient level. After the removal, the signal was backward reconstructed by use of an inverse wavelet transformation.

#### 2.1.4. Feature Extractions

In this study, we compared three feature extraction methods: (i) power spectral density (PSD); (ii) autoregressive (AR) modeling; (iii) multiscale entropy (MSE). It is well established that EEG activity can be analyzed by frequency-domain features. The PSD is the most common feature calculated using fast Fourier transform (FFT). Previous studies have shown that PSD is strongly related to drowsiness and KSS scores [19,40]. To obtain the PSD, a windows function was used to reduce the effects of leakage that occur during FFT. We used a Blackman window to obtain an effective resolution [46]. Use of this window function results in a lower frequency resolution but a wider dynamic range. We initially grouped the frequencies as 1–4 Hz, 4–8 Hz, 8–10 Hz, 10–12 Hz, 12–14 Hz, 14–26 Hz, 26–40 Hz, 40–49 Hz, 51–65 Hz, 65–80 Hz, and 80–100 Hz. The upper range of the beta band is considered to be 26 Hz. The range beyond 26 Hz is generally defined as the gamma band; however, the definition of the gamma band varies [47,48]. To account for this, we extended our range to 100 Hz and divided the resulting upper range into five segments.

Although it is well established that the PSD is strongly related to the KSS score, other feature extraction methods related to sleep or fatigue should be compared. In recent studies, an autoregressive (AR) model has been applied for EEG analysis as a spectrum transformer instead of the FFT [32,33,49]. We used AR model parameters as a feature. The parameters can be calculated by using the Yule–Walker equation. The advantage of using an AR model is that it performs better for short-wave data; however, the difficulty in selecting this method is in deciding the number of order to be used in the model. In this study, we selected five as the number of order to be used, in accordance with a previous study about fatigue classification [33].

Entropy-based feature extraction has also been used for EEG analysis of sleep- and fatigue-related studies [32,50,51,52]. Entropy is used to quantify the complexity of time-series data such as that of the EEG signal. Approximate entropy (ApEn) and sample entropy (SampEn) are representative methods of entropy and have been widely used in previous studies. For single-channel EEG, usage of MSE as a feature has been proposed [53,54]. The MSE computes SampEn with different scales and measures the complexity of the time-series data with respect to multiple temporal scales. In this study, we selected the parameters m=2 and r=0.15×SD, in line with previous results indicating that m=1 or 2 and r=0.1×SD−0.25×SD provide good statistical validity for SampEn [55,56].

Frequency feature selection is an important problem in the detection of drowsiness. The round-robin method and optimization methods, such as genetic algorithms (GA), can be applied. Because we selected the hyperparameters of the SVM in this study, a selection method that is not affected by the SVM is required. Therefore, we used step-wise linear discriminant analysis (SWLDA), which has been widely used in BCI studies [28,57] for feature selection. The SWLDA extracts features using both forward and backward steps. Initially, *p*-values are calculated from an F-statistic for each feature. New features are added when the *p*-value of a given feature falls below the input threshold (*p*-value < 0.1). Each time a new feature is added, *p*-values are recalculated and the features that are no longer significant are removed (*p*-value > 0.15). This powerful linear method can reduce time costs and prevent the overfitting of feature selection. In this study, we compared the PSD selected by the SWLDA and the PSD of the theta and alpha band that was selected following previous studies [1,26,27].

EEG readouts change by the second regardless of any fluctuations in drowsiness. Time-averaging is an effective means of reducing changes in the EEG signal that are not related to drowsiness. Through the use of time-averaging, we translated our EEG data into a more stable form. We used a 10 s time window with a 10 s shift. The 60 s EEG recordings were compressed into six samples. To prevent overfitting, a scaling method can also be useful. In this study, we used the robust scaler methodology. Scaling methods transform data into a common scale by adjusting the lower limit of the data. The robust scaler methodology calculates the median and the normalized interquartile range (NIQR) of the data set using the following equations:

(1) NIQR≈IQR×0.7413

(2) $$z_{i}=\frac{\left(x_{i}-x_{m}\right)}{NIQR},$$

where $z_{i}$ is the scaled vector and $x_{m}$ is the median of the original data set, $x_{i}$. NIQR is the normalized interquartile range of the original data set. The interquartile range (IQR) is generally calculated from the 25th quantile to the 75th quantile, however, in this study we used the 35th and 65th quantiles. The median was chosen for use because it ignores the shape of the distribution. Because the NIQR is not affected by outliers, robust scaling can be applied to the EEG signal measured by dry electrodes, the distribution of which is unpredictable.

#### 2.1.5. Pattern Recognition

Scores obtained from the KSS took the form of integers ranging from 1 to 9. We developed the drowsiness detection model using the support vector machine (SVM), which has been used for estimation of drowsiness from EEG data with high accuracy [7,25]. Through the use of a kernel function, the SVM can classify data nonlinearly. In this study, it was used for classifying two classes: the KSS scores falling below a threshold and those falling above that threshold. We used two classes in our analysis because classification of more than two classes with EEG data obtained only from Fp1–A1 is much more complicated, and our aim was to compare performance using the binary classification criteria mentioned in Section 1. The SVM classifies two classes by minimizing the evaluation function using the following equation,

(3) $$\min_{w}\frac{1}{2}{\left\|w\right\|}^{2}+C\sum_{i=1}^{N}\xi_{i}$$

(4) $$s.t.\left\{\begin{array}{c} y_{i}\left(w^{T}x_{i}+b\right)\geq 1-\xi_{i}\\ \xi_{i}\geq 0 \end{array}\right.$$

where $x_{i}$ is the feature vector of the learning data, $y_{i}$ is the label of the training data, and *C* is the regularization constant.

For solving the above equations, kernel functions are used to map features into a high-dimensional space. In previous studies, the radial basis function (RBF) kernel has been used due to its high accuracy in EEG studies [57,58]. The kernel is defined by the following equation:

(5) $$K\left(x_{i},x_{j}\right)=exp(-\gamma \|x_{i}-x_{j}{\|}^{2}),\gamma >0.$$

We used the RBF kernel for the SVM in this study. *C* and γ are tuning hyperparameters and were determined using a grid search. To conduct a grid search, an evaluation function first needs to be defined. We used the classification accuracy for the SVM.

To prevent overfitting of our model during learning, we used 10-fold cross-validation in this study. To carry out this method of validation, the data set was randomly split into 10 subgroups of equal size, 9 of which were used for pattern learning and the remaining of which were used for testing and calculating output scores from the evaluation function. This process was repeated 10 times until all subsamples were suitable for use during testing. *C* was changed from $2^{-2}$ to $2^{11}$ and γ was changed from $2^{-10}$ to $2^{3}$ in accordance with previous studies [59,60]. To tune hyperparameters and evaluate accuracy without overfitting our model, we used 10-fold cross-validation as nested cross-validation. The training data formed by 10-fold cross-validation was split by further 10-fold cross-validation and used for optimization of our hyperparameters. Incorporating these hyperparameters, our exterior test data were used for the prediction and calculation of indicators, including classification accuracy. In this study, true positive (TP) refers to how accurately drowsy data is classified as drowsy data. True negative (TN) refers to how accurately alert data is classified as alert data. False positive (FP) indicates the percentage of data incorrectly identified as drowsy, and false negative (FN) indicates the percentage of data incorrectly identified as alert. Precision, sensitivity, specificity, classification accuracy (Acc), and F-measure are calculated using the following equations:

(6) $$Precision=\frac{TP}{TP+FP}$$

(7) $$Sensitivity\left(Recall\right)=\frac{TP}{TP+FN}$$

(8) $$Specificity=\frac{TN}{TN+FP}$$

(9) $$Acc=\frac{TP+TN}{TP+FP+FN+TN}$$

(10) $$F-measure=2\times \frac{precision\times recall}{precision+recall}$$

### 2.2. Results

**Figure 3:**
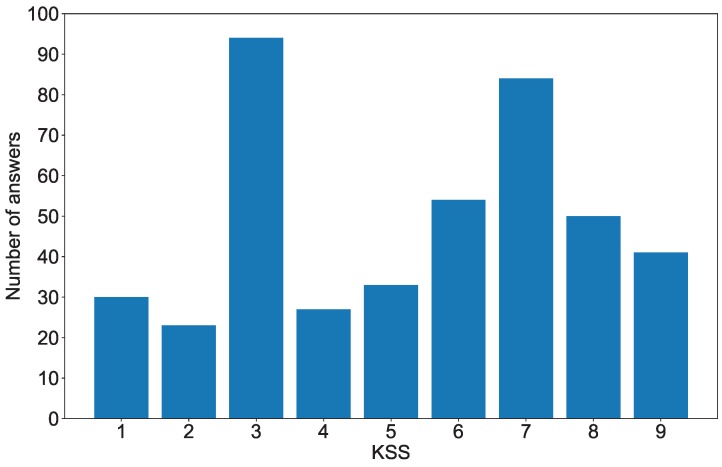
A histogram of Karolinska Sleepiness Scale (KSS) scores and number of answers.

A total of 435 data sets (three times a day over 5 days with 29 subjects) were analyzed for the development of our model. The 60 s data were divided by a moving averaging, and a total of 2610 samples were used for training the SVM. To compensate for EEG data lacking a corresponding KSS score or containing errors and equalize the number of data sets across subjects, we excluded 2 days of data from each subject. The distribution of the recorded KSS scores are shown in Figure 3. To assess performance of the features and classification methods we used without overfitting, we used 10-fold cross-validation. Determining a well-defined threshold is an important factor for developing an effective drowsiness detection system. In this study, we used two kinds of thresholds: (i) one threshold, A, to define two classes (class1 < A, A < class2), (ii) two thresholds, A and B, to form the two classes (class1 < A, B < class2).

**Figure 4:**
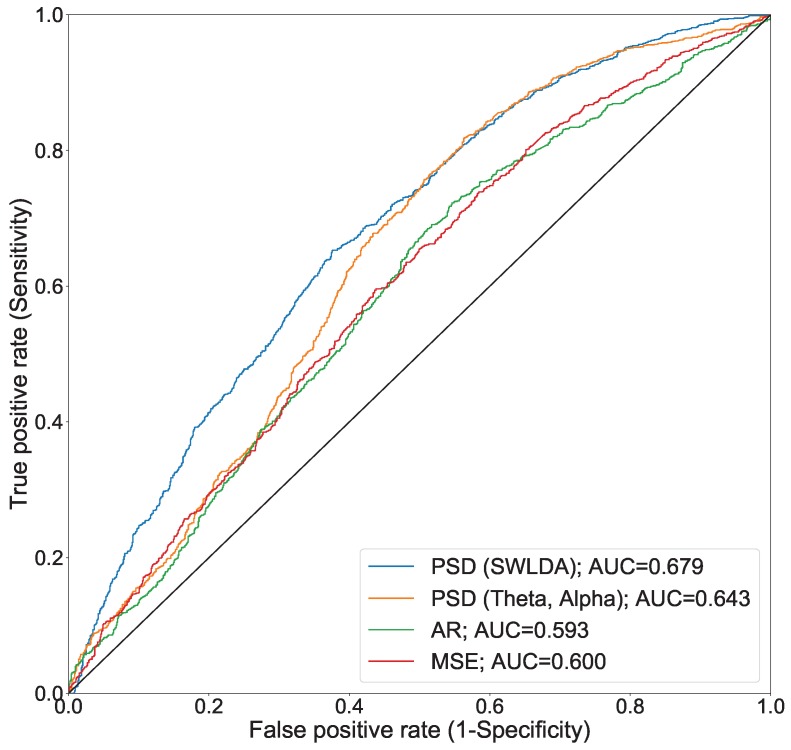
Receiver operating characteristic (ROC) curves for the autoregressive (AR) model support vector machine (SVM) and step-wise linear discriminant analysis (SWLDA) SVM.

To assess the developed model, we compared feature extraction methods with one threshold by using the receiver operating characteristic (ROC) curve shown in Figure 4. The ROC curve uses the true positive rate (sensitivity) and false positive rate (1-specificity) calculated using 10-fold cross-validation with several thresholds. The area under the curve (AUC) was calculated by an average of the approximations under the ROC curves. Figure 4 shows which of the plots of the PSD features had the greatest upper-left curve when compared with AR and MSE. PSD (SWLDA) resulted in an AUC of 0.679. PSD (Theta, Alpha) resulted in an AUC of 0.643. They were greater than those produced by AR, which resulted in an AUC of 0.593, as well as MSE, which had an AUC of 0.600. Table 1 shows the number of samples labeled as drowsy and alert with several different thresholds. A total of 1354 samples of drowsy-state data and 1037 samples of alert-state data were used for classification when the threshold was set to five, which is the centermost point of the KSS. Table 2 shows the precision, sensitivity, specificity, binary-classification accuracy (Acc), and F-measure for the classification. The two features of PSD resulted in a greater Acc and F-measure compared with AR and MSE. There was little difference between PSD (SWLDA) and PSD (Theta, Alpha). PSD (SWLDA) resulted in an Acc of 64.3% and an F-measure of 72.8%. PSD (Theta, Alpha) resulted in an Acc of 65.0% and an F-measure of 73.7%. The sensitivity of PSD (SWLDA), with a value of 83.9%, was lower than that of PSD (Theta, Alpha), which had a value of 86.6%. The specificity of PSD (SWLDA), with 38.8%, was greater than that of PSD (Theta, Alpha), with 36.6%.

We assumed that the labeled data close to middle of the KSS did not indicate strong alertness or drowsiness. To account for this, we used two threshold methods to divide the data into two classes (alert vs. drowsy). Firstly, KSS scores of 4 and 6 were used as thresholds (alert < 4 and 6 < drowsy). A total of 1038 samples of drowsy-state data and 875 samples of alert-state data were used for classification, as shown in Table 1. In this condition, PSD with SWLDA had the greatest Acc, with a value of 67.2% in three features, as shown in Table 3. The F-measure was 71.3%. The sensitivity of PSD (SWLDA), with 74.3%, was lower than that of PSD (Theta, Alpha) with 85.4%. Contrary to this result, the specificity of PSD (SWLDA), with 58.7%, was greater than that of PSD (Theta, Alpha), which had a value of only 41.7%. A total of 538 samples of drowsy-state data and 314 samples of alert-state data were used for classification when the KSS thresholds were set to 3 and 7, as shown in Table 1. Table 4 shows the results. The highest accuracy was attained by PSD with SWLDA, with an Acc value of 72.7% and an F-measure of 80.1%. The PSD (SWLDA) also resulted in the best precision, with a score of 73.5% and a specificity of 45.2%. Only the sensitivity of PSD (SWLDA), with a value of 45.2%, was lower than PSD (Theta, Alpha), but the difference between the two sensitivities was small when compared with the results when the threshold was set to 4 and 6. In addition to 10-fold cross-validation, leave-one-subject-out cross-validation (LOSOCV) is important to assess classification performance. In the condition where the thresholds were set to 3 and 7 and classification performance was assessed by LOSOCV, the accuracy of PSD using the LOSOCV was higher than the accuracy using other features. The PSD (SWLDA) resulted in a sensitivity of 88.3%, a specificity of 32.2%, and an Acc of 66.1%. The PSD (Theta, Alpha) resulted in a sensitivity of 84.2%, a specificity of 38.2%, and an Acc of 66.0%.

## 3. Practical Experiment

### 3.1. Experimental Procedure

The practical experiment was approved by the Ethical Review Committee of Dentsu ScienceJam Inc. (approval number 005). Twenty healthy subjects took part in our experiment (8 females and 12 males, mean age = 34.2, SD = 10.2). They did not have any mental diseases or psychological disorders. All subjects were non-smokers and were asked to abstain entirely from intake of caffeine, nicotine, and alcohol after 21:00 on the day before the experiment. We explained the details of the experiment so that the subjects had a full understanding of the procedure. Before taking part in the experiment, subjects completed an informed consent form. We made it clear that they had a right to withdraw from the experiment at any time if it caused them any discomfort.

**Figure 5:**
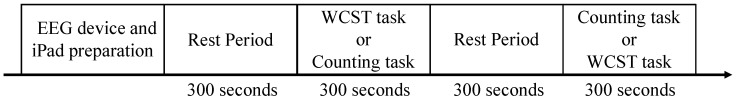
The practical experiment procedure, during which EEG recordings of participants were obtained throughout the simple counting task and Wisconsin Card Sorting Test (WCST) task.

The experimental procedure we followed is depicted in Figure 5. The experiment consisted of two tasks, both of which were completed by all participants. The first task consisted of counting from 1 to 300 (simple counting task) with open eyes and was intended to evoke drowsiness in the participants. The second task was the Wisconsin Card Sorting Test (WCST) [61,62]. In the WCST, participants are presented with a number of cards on a display and must match the cards based on either shape, color, or number, though they are not told which feature to use. The feature that participants must use to sort the cards changes after a random number of trials, promoting attentiveness by forcing participants to shift sorting rules. After each selection, they are given feedback as to whether or not their selection was correct, though they are not informed why their selection was or was not correct. Although the duration of time taken for each round varies depending on the participant, we stopped the task after 300 s had elapsed because the purpose of the task was to keep participants wakeful rather than obtain completed WCST responses. The WCST task was conducted on a PC. The order of the two conditions was randomized, with half of participants beginning with the counting task and the other half beginning with the WCST.

**Figure 6:**
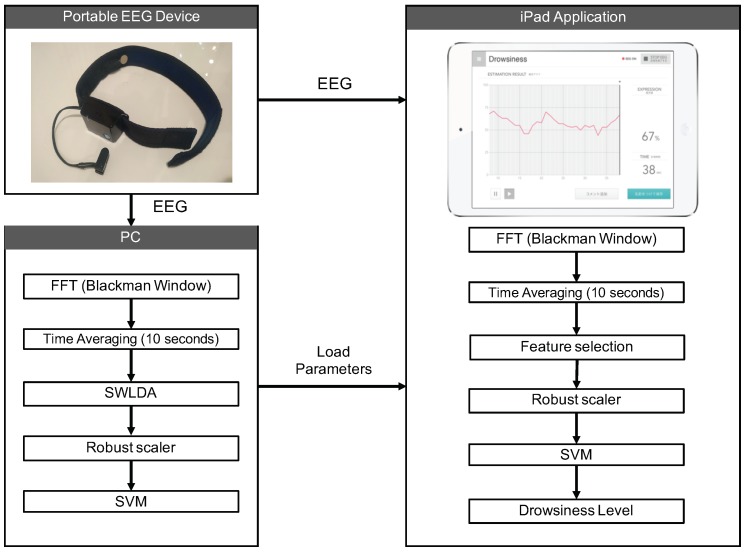
Flow diagram of the drowsiness detection system. Parameter learning was performed on a PC. The selected parameters were loaded onto the iPad and show the predicted drowsiness level.

EEG recording was conducted using the iPad application shown in Figure 6. The model developed in Section 2 was used in the application. As shown in the results of Table 4, PSD with SWLDA was used as a feature. The thresholds 3 and 7 were used to divide data into two classes. The frequency ranges of PSD were selected as 1–4 Hz 4–8 Hz, 10–12 Hz, and 12–14 Hz using the EEG data recorded in Section 2. The hyperparameters of the SVM were determined using a grid search. Again, both the range and shift of time-averaging was 10 s. The robust scaling thresholds were reused for this experiment. As indicated by the results of Section 2, classification accuracy was higher when using PSD, but the optimal frequency features were not clearly indicated. Therefore, both frequency features were applied to the recorded EEG data. We conducted SWLDA for the application online throughout the experiment. The theta–alpha features were applied to the recorded data offline after the experiment was completed.

### 3.2. Results

**Figure 7:**
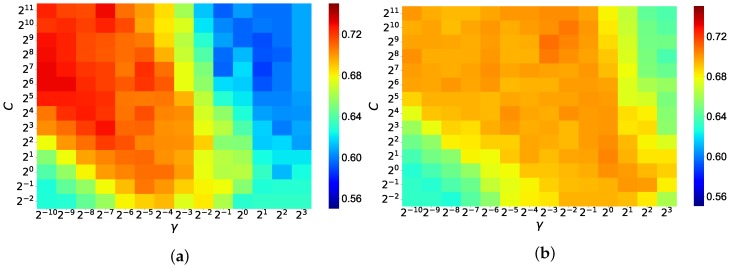
(**a**) Grid search result of the SVM. Classification accuracies are denoted by color. The optimal hyperparameters were $C=2^{6}$ and $\gamma =2^{-9}$; (**b**) grid search result of the SVM. Classification accuracies are denoted by color. The optimal hyperparameters were $C=2^{9}$ and $\gamma =2^{-3}$.

**Figure 8:**
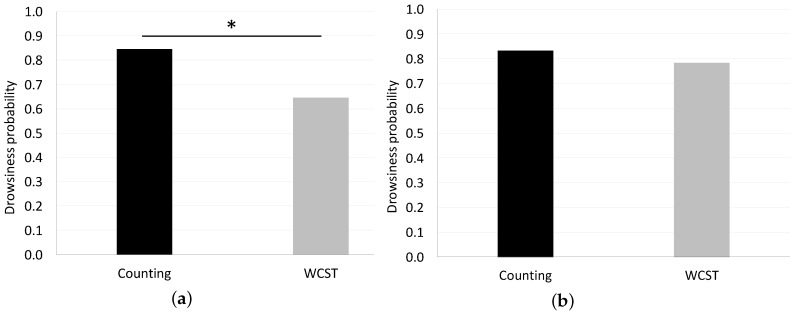
(**a**) The average drowsiness probability of the 20 subjects, as generated by the PSD (SWLDA) and SVM. Scores were significantly higher during the counting task (0.85 vs. 0.65, * *p* < 0.05); (**b**) the average drowsiness probability of the 20 subjects, as generated by the PSD (Theta, Alpha) and SVM. The average score was higher during the counting task (0.83 vs. 0.78).

The results of the grid searches are displayed as a heatmap in Figure 7. Colors denote classification accuracies in Figure 7; the colors change progressively as hyperparameters are shifted. The accuracy of PSD (SWLDA) was larger overall than PSD (Theta, Alpha) in the grid search progress. PSD (SWLDA) had a stronger dependence on hyperparameters than PSD (Theta, Alpha) due to the fact that the red-colored area was biased by a lower γ parameter. In this practical experiment, the EEG recordings were transformed into drowsiness level through the use of the SVM. The SVM produces binary output data made up of zeroes and ones. Each data package of 300 s was averaged using 10 s windows with a 10 s shift, resulting in 30 drowsiness level samples for each data recording. In order to score each package of recorded data, we averaged the 30 drowsiness level samples and transformed them into a drowsiness probability, which was 1.00 when all outputs of the four samples were one and 0.00 when all outputs were zero. The average of the drowsiness probabilities calculated by the PSD (SWLDA) with SVM for the simple counting task was higher than the average of the scores generated for the WCST (0.85 vs. 0.65, * *p* < 0.05), as shown in Figure 8a. The average of the estimated drowsiness probabilities generated by the PSD (Theta, Alpha) with SVM for the counting task was higher than the generated scores for the WCST (0.83 vs. 0.78), as shown in Figure 8b. Both results produced an average estimate of drowsiness level for the counting task that was higher than that of the WCST. Significant differences are indicated by starred *p*-values calculated by a *t*-test. The results produced by the PSD (SWLDA) with SVM indicated a greater and more significant difference between the two conditions than the results produced by the PSD (Theta, Alpha).

## 4. Discussion

### 4.1. Accuracy of Drowsiness Detection

In this study, we obtained prefrontal single-channel EEG recordings and KSS scores from 29 participants and developed a system to detect drowsiness using EEG. Two criteria were used in evaluating the accuracy of our system: classification accuracy in the development of our model and the significance of the difference between conditions in the case of our practical experiment. For classification accuracy, binary classification accuracy (alert/drowsy) resulted in a value of 72.7%, as determined by 10-fold cross-validation using PSD (SWLDA) with SVM. In the comparison of extraction methods, the PSD (SWLDA) resulted in an AUC of 0.679. PSD (Theta, Alpha) resulted in an AUC of 0.643. They were greater than those produced by AR, which resulted in an AUC of 0.593, as well as MSE, which had an AUC of 0.600. We presume that the result indicating PSD had a higher AUC depends on the EEG recording situation and the number of EEG channels. We collected EEG measurements not while driving, but during resting states. Kaida et al. evaluated the relationship between PSD and the KSS when subjects were in a state of rest [19]. Moreover, all fatigue detection studies using an AR model have utilized multichannel EEG. Due to this fact, they could obtain much more detailed information than was available using our device.

As mentioned in Section 1, our aim was to attain an accuracy of 70% or higher for binary classification. We recognize that this criterion is lower than that used in previous studies, which generally detect drowsiness with over 90% accuracy for binary classification [11]. However, a classification accuracy of over 70% is acceptable given the constraints imposed by our device. There is a clear trade-off between usability and accuracy in developing portable, cost-effective devices such as ours. We only made use of the measurement position Fp1–A1, which only weakly reflects human drowsiness and sleepiness [40]. In contrast to previous studies that have used multichannel devices, we had to detect the drowsiness level within a single channel. As demonstrated in our practical experiment, our system is capable of significantly discriminating between wakeful and drowsy states in a task-based setting given an adequate amount of sample data.

### 4.2. Selected Features and Parameters

Previous studies have reported that the theta and alpha bands of EEG are correlated with human drowsiness [4,19,20,21,22]. In this study, we used both SWLDA-based frequency feature selections and theta–alpha bands. As indicated by the results presented in Table 2, PSD (Theta, Alpha) resulted in a higher performance than PSD (SWLDA). This stands in stark contrast to the results presented in Figure 4, Table 3 and Table 4. However, the specificity of PSD (SWLDA) in all conditions was greater than that of PSD (Theta, Alpha). Moreover, the ROC curve of PSD (SWLDA) was located in the upper-left in lower sensitivity and 1-specificity compared with that of PSD (Theta, Alpha). These results demonstrate that PSD with SWLDA is more robust when using biased sample data in which the number of drowsy samples is greater than the number of alert samples.

Using SWLDA, we selected the frequency ranges 1–4 Hz 4–8 Hz, 10–12 Hz, and 12–14 Hz for use in the practical study. The 1–4 Hz range falls in the delta band, 4–8 Hz falls in the theta band, and 10–12 Hz and 12–14 Hz are part of alpha band. Although the alpha and theta bands are known to be correlated with the KSS, a previous study reported that the delta band is strongly correlated with fatigue [63]. Because the KSS is also used to measure fatigue [64], the selection of the delta band is appropriate from the viewpoint of estimating the KSS. In fact, the features including that frequency range showed a higher level of performance. However, this result may also indicate that the model is predicting fatigue level. In this study, both the counting task and WCST may induce fatigue in participants. Taking this into account, our model may reflect drowsiness rather than fatigue. Further support of the interpretation that our model produces stronger estimates for drowsiness than fatigue is provided by comparison with a recent study of fatigue classification [33]. The authors of this study demonstrated that the AR model achieved better classification results when compared with the results produced using PSD features in contrast to the results of our study.

In Figure 7 shows the distribution of classification accuracies when changing the hyperparameters of the SVM. Although overfitting occurs at extreme peaks in the heatmap, distributions are not sharp around the best hyperparameters. This indicates that our portable system continues to detect drowsiness with similar performance when the learning data is updated and the best hyperparameters are changed. For the windows function of the FFT, we used the Blackman window, though the Hamming window is more common. We used the Hamming window as well, but the calculated performances were lower than those produced by the Blackman window, so those results are not reported in this study. However, the difference between the two was slight, suggesting that the type of window function used does not have a large effect on performance during drowsiness detection. For the robust scaler, we used the 35th and 65th quantile instead of the 25th and 75th quantile. We tried applying these common quantiles as well; however, they did not show higher accuracies when compared with the quantiles used in our system. The signal quality of the data obtained from the low-priced, dry EEG we used is low. Because of this, the NIQR should be calculated using the 35th and 65th quantile to reduce the effects of outliers.

### 4.3. Limitations

In this study, the PSD resulted in the best performance as a feature for drowsiness detection. In contrast to our results, recent studies focusing on sleep stage and driver fatigue have obtained higher performance with the use of entropy features or AR modeling in their evaluations [32,33,50]. There are three reasons for this difference: (i) measurement point, (ii) drowsiness rating method and (iii) EEG recording situation. Our study used only the Fp1–A1 measurement point. That area only weakly reflects sleepiness. We used KSS scores as ratings of drowsiness level. Power spectral density has been demonstrated in previous studies to be strongly related to KSS scores and measured human drowsiness [19,40]. Most recent studies have recorded EEGs while subjects were driving. We recorded the EEG when the subjects were in a resting state. When we apply our model to driving states, the results may differ. Therefore, we recommend this comparison be conducted in future studies.

Studies using multichannel EEG devices have shown higher accuracy for drowsiness detection when compared with the performance of our portable system [65,66]. When applying drowsiness detection techniques using EEG for safety-related situations, such as driving, multichannel EEG devices should be applied because our method’s accuracy of around 70% is not high enough to be suitable for safety purposes. Nevertheless, the accuracy achieved by our technique may be suitable for non-safety-related situations. Previous studies have used EEG for TV commercial evaluation and real-time inference of engagement in the classroom [67,68]. After collecting enough data for analysis, our portable system can yield significant results, as evaluated in Section 3. The technique we developed can be applied to rating movies, concerts, and other activities that have the potential to evoke drowsiness. Since our portable systems are user-friendly and inexpensive, they can readily be used within practical situations.

For the classification method, we used the SVM, which uses a support vector, as classifier. A support vector regression (SVR) also makes use of a support vector and can be trained for estimating drowsiness scores as a continuous value. We also used the SVR for KSS estimation; however, the estimation accuracy was not high. The EEG data recorded from our low-priced device has not yet been able to accurately estimate KSS scores using regression methods. In addition to the support vector-based classification, neural network-based classifiers have also been proposed as a method of classification [33,69]. We used the SVM in this study because the difference in accuracy between the SVM and neural networks is not significantly different, and drowsiness detection with KSS scores has been performed by SVM in recent studies [11,25]. We recommend that future studies explicitly compare neural network-based methods with the methods used in this study.

## 5. Conclusions

In this paper, we developed a portable system capable of detecting human drowsiness through use of a single-channel, low-priced EEG device. We collected EEG recordings while subjects were in a resting state. To improve drowsiness detection, results obtained using PSD, AR modeling, and MSE were compared. The SVM was used to classify EEG data as representing alert or drowsy states, and its hyperparameters were subsequently optimized. These techniques allowed our low-priced device to detect drowsiness levels with a classification accuracy of 72.7% (sensitivity 88.7% and specificity of 45.2%) for binary classification (alert/drowsy). To assure the performance of our system in practical settings using methods of statistical interpretation, our developed system was applied while users took part in both a simple counting task and the WCST. The results of this experiment indicated a significant difference between the two tasks in participant level of drowsiness.

There is a trade-off between usability and accuracy in the development of measurement techniques such as ours. Though our technique has the potential to be more practical and accessible than conventional EEG drowsiness measurement systems, this practicality came at the cost of reduced accuracy. Previous studies that have demonstrated higher accuracy in drowsiness detection using multichannel EEG should be favored as a basis for purposes requiring a high degree of safety, while our technique is more suited to budget-limited, non-safety-related purposes. For example, our technique could be applied to use students’ drowsiness levels to assess classroom engagement in schools. It could be applied in a similar way to assess movies and products. By evaluating and improving our system by applying it to a wider range of situations, the reliability of our system will continue to improve. This, in turn, will increase the range of practical situations that our system can be applied to.

References

## Figures and Tables

**Table 1:** The number of KSS samples labeled as drowsy and alert using three different thresholds.

Criteria	Thresholds		
	5	4 & 6	3 & 7
KSS Sample (Drowsy)	1354	1038	538
KSS Sample (Alert)	1037	875	314

**Table 2:** Performance summary of alert vs. drowsy classifications when the threshold for the two classes was set to 5.

Criteria	PSD (SWLDA)	PSD (Theta, Alpha)	AR	MSE
TP	1136	1172	1077	1120
FP	635	657	690	725
FN	218	182	277	234
TN	402	380	347	312
Precision (%)	64.1	64.1	61.0	60.7
Sensitivity (%)	83.9	86.6	79.5	82.7
Specificity (%)	38.8	36.6	33.5	30.1
Acc (%)	64.3	64.9	59.6	59.9
F-measure (%)	72.7	73.6	69.0	70.0

**Table 3:** Performance summary of alert vs. drowsy classifications when the thresholds for the two classes were set to 4 and 6.

Criteria	PSD (SWLDA)	PSD (Theta, Alpha)	AR	MSE
TP	771	886	780	689
FP	361	510	579	507
FN	267	152	258	349
TN	514	365	296	368
Precision (%)	68.1	63.5	57.4	57.6
Sensitivity (%)	74.3	85.4	75.1	66.4
Specificity (%)	58.7	41.7	33.8	42.1
Acc (%)	67.2	65.4	56.2	55.3
F-measure (%)	71.1	72.8	65.1	61.7

**Table 4:** Performance summary of alert vs. drowsy classifications when the thresholds for the two classes were set to 3 and 7.

Criteria	PSD (SWLDA)	PSD (Theta, Alpha)	AR	MSE
TP	477	492	503	461
FP	172	209	245	210
FN	61	46	35	77
TN	142	105	69	104
Precision (%)	73.5	70.2	67.2	68.7
Sensitivity (%)	88.7	91.4	93.5	85.7
Specificity (%)	45.2	33.4	22.0	33.1
Acc (%)	72.7	70.1	67.1	66.3
F-measure (%)	80.4	79.4	78.2	76.3
